# The Y-Health Prospective Study of Physical Health in Young People in Mental Health Inpatient Units

**DOI:** 10.1192/bjo.2025.10125

**Published:** 2025-06-20

**Authors:** Rebekah Carney, Shermin Imran, Olorunleke Erunkulu

**Affiliations:** Greater Manchester Mental Health NHS Foundation Trust, Greater Manchester, United Kingdom

## Abstract

**Aims:** To explore the physical health of YP admitted to adolescent inpatient mental health units and reflect on any differences over the following 6 months.

Research Questions: 1. To assess physical health of young people upon admission to adolescent inpatient services (cardiovascular risk factors e.g. BMI, blood pressure, blood glucose and lipids). 2. To assess current lifestyle behaviours of young people upon admission to adolescent inpatient wards (e.g. physical activity, diet, smoking rates). 3. To assess changes in physical health/lifestyle 3 months and 6 months post-admission. 4. To understand the impact of inpatient care environment on lifestyle behaviours and physical health of adolescents admitted to inpatient units. 5. To understand the experiences and beliefs about physical health in adolescents admitted to inpatient units. 6. To establish the feasibility of monitoring physical health in a cohort of young people upon admission to an adolescent inpatient unit.

**Methods:** We aimed to recruit young people aged 14+ from each participating site within 6 weeks of admission to the unit. The young person needed to be able to give informed consent and be well enough to take part (severe anorexia/eating disorder excluded). Physical and mental health assessments were completed by a researcher in conjunction with the clinical team. Assessments completed at three time points: Baseline on admission; 3 months post admission; 6 months post admission. Participants given £10 voucher at each timepoint as a thank you (total £30).

Measures collected included: Demographic information, e.g. age, gender, ethnicity, education, diagnoses, previous admissions, medication, length of admission; Physical Health Outcomes, e.g. BMI (centiles), BP, routinely collected blood tests (random glucose, lipids, etc), ECG; Behavioural Outcomes, e.g. physical activity levels, smoking status, diet, physical fitness (six-minute walk & questionnaire), substance use, comorbid physical health disorders and concurrent treatments; Mental Health Outcomes, e.g. Health of the Nation Outcome Scales for Children and Adolescents (HONOSCA), World Health Organization Wellbeing Index (WHO-WI).

**Results:** Physical health outcome (Weight): Baseline – 64.5. 3 months – 67.7. 6 months – 69.3. Behavioural outcome: Low levels of physical activity (average 20 mins sport and 1 hour walking per day); High levels of sedentary behaviour; Most common substances used were alcohol (n=11, 44%), tobacco (n=10, 40%) and cannabis (n=6, 25%); Most YP self-reported average fitness levels; Consumed on average 1.8 meals per day (ranged from 1–5).

HONOSCA outcome: 80% lack of concentration (68% severe); 75% self-harmed; 56% difficulties with relationships at home (30% severe); 88% anxious or low mood (44% severe); 64% impairments with educational ability; 64% stopped attending education.

Qualitative interviews (thematic analysis): Outcomes on Young peoples knowledge, Autonomy, environment, sources of support, independence and facilitators.

**Conclusion:** Young people on CAMHS inpatient units have multiple factors affecting their physical health; Already showing some signs of compromised physical health, likely to worsen; Observed lots of challenges with transitory care and barriers to following people up after discharge; Future work will focus on breaking down some of the barriers experienced to living well; Working on refining a physical health intervention.

